# Dimethyl Fumarate and Its Esters: A Drug with Broad Clinical Utility?

**DOI:** 10.3390/ph13100306

**Published:** 2020-10-13

**Authors:** Stephanie Kourakis, Cara A. Timpani, Judy B. de Haan, Nuri Gueven, Dirk Fischer, Emma Rybalka

**Affiliations:** 1 College of Health and Biomedicine, Victoria University, Melbourne, VIC 8001, Australia; stephanie.kourakis@live.vu.edu.au; 2 Institute for Health and Sport, Victoria University, Melbourne, VIC 8001, Australia; cara.timpani@vu.edu.au; 3 Australian Institute for Musculoskeletal Science, Victoria University, St Albans, VIC 3021, Australia; 4 Oxidative Stress Laboratory, Baker Heart and Diabetes Institute, Basic Science Domain, Melbourne, VIC 3004, Australia; judy.dehaan@baker.edu.au; 5 Department of Physiology, Anatomy and Microbiology, La Trobe University, Melbourne, VIC 3083, Australia; 6 School of Pharmacy and Pharmacology, University of Tasmania, Hobart, TAS 7005, Australia; Nuri.Guven@utas.edu.au; 7 Division of Developmental- and Neuropediatrics, University Children’s Hospital Basel, University of Basel, 4056 Basel, Switzerland; Dirk.Fischer@ukbb.ch

**Keywords:** clinical application, dimethyl fumarate, disease, fumaric acid esters, oxidative stress, inflammation, Nrf2, disease

## Abstract

Fumaric acid esters (FAEs) are small molecules with anti-oxidative, anti-inflammatory and immune-modulating effects. Dimethyl fumarate (DMF) is the best characterised FAE and is approved and registered for the treatment of psoriasis and Relapsing-Remitting Multiple Sclerosis (RRMS). Psoriasis and RRMS share an immune-mediated aetiology, driven by severe inflammation and oxidative stress. DMF, as well as monomethyl fumarate and diroximel fumarate, are commonly prescribed first-line agents with favourable safety and efficacy profiles. The potential benefits of FAEs against other diseases that appear pathogenically different but share the pathologies of oxidative stress and inflammation are currently investigated.

## 1. Introduction

Dimethyl fumarate (DMF) is a methyl ester of fumaric acid (FA) (i.e., an FA ester (FAE)) registered for the treatment of relapsing forms of Multiple Sclerosis (MS) [1,2] and psoriasis [3,4]. As an α, β-unsaturated carboxylic ester generated by reacting FA with methanol in the presence of sulfuric acid, DMF notably stimulates mitochondrial tricarboxylic acid (TCA) cycle activity and adenosine triphosphate (ATP) production [5]. Whilst FA alone is poorly absorbed in the gastrointestinal (GI) tract, DMF and its active metabolite monomethyl fumarate (MMF) to which it is almost entirely converted in the gut [6], have robust bioavailability (for pharmacokinetics data see [6]) and exert beneficial effects in diseases characterised by inflammation [7,8,9], neurodegeneration [10,11] and toxic oxidative stress [9,12]. A modernized version of DMF has recently been developed in diroximel fumarate (DRF), which has comparable efficacy against MS to DMF but with fewer side effects [13].

The therapeutic efficacy of FAEs appears to be mediated through the activation of the nuclear factor erythroid 2-related factor 2 (Nrf2) transcriptional pathway [14,15,16] as well as interaction with the anti-inflammatory hydroxycarboxylic acid receptor 2 (HCAR2) [17,18]. The Nrf2 pathway is considered a critical cellular defence system that responds to potentially toxic stimuli via the upregulation of antioxidant and Phase II cytoprotective response genes [19,20]. A number of Nrf2 inducing drugs have been developed and tested in clinical [1,21,22,23,24] and pre-clinical [25,26,27,28,29] studies over the past decade and many have demonstrated potential for clinical progression. These studies highlight the broad therapeutic utility of targeted Nrf2 activation against a variety of diseases (summarised in Table 1). In addition to the activation of Nrf2, MMF also interacts with HCAR2, which strongly modulates anti-inflammatory activity, particularly in primary and accessory immune cells [30]. By reversing mitochondrial TCA cycle flux, there is novel evidence that FAEs might exert at least a portion of their immunomodulatory action through generating succinyl-carnitine esters or other metabolites as well [31,32]. The collective Nrf2-inducing and immuno-modulatory properties of DMF and other FAEs, have demonstrated therapeutic efficacy across multiple body systems such as the central nervous [10,33], cardiovascular [34,35], gastrointestinal [7,36,37], immune [5,38] and integumentary [39,40] systems. Thus, the prospective clinical impact of DMF and its esters is extensive.

This review discusses the efficacy and side-effect profiles of FAEs with reference to reported data from completed and ongoing clinical and pre-clinical trials and postulates new clinical indications.

## 2. Approved Indications for Dimethyl Fumarate (DMF)

Oxidative stress occurs when oxidant production (i.e., reactive oxygen species (ROS)) exceeds the capacity of endogenous antioxidants to scavenge and detoxify ROS, and generally leads to oxidative cellular damage [41,42]. ROS can oxidise biomolecules and structurally modify proteins and DNA to trigger signalling cascades that both cause and progress tissue damage and inflammation [41]. Although inflammation is an ostensibly beneficial response to tissue injury in which immune cells are recruited to digest necrotic tissue and promote new tissue regeneration, chronic inflammation potentiates oxidative stress and tissue injury through persistent inflammatory cytokine release [43]. In response to cytokines, immune cells generate more ROS, escalating oxidative stress and insult to tissues [43]. This paradoxical interrelation between oxidative stress and inflammation has been linked to many chronic inflammatory diseases including psoriasis and MS. The efficacy of FAEs is proposed to be based on their dual antioxidant and anti-inflammatory effects, which justifies the global approval of FAE drugs for both indications.

### 2.1. DMF for Psoriasis

Psoriasis is a non-contagious, chronic, multi-system inflammatory skin disease characterised by recurrent episodes of red, scaly skin plaques that are distinctively demarcated from the normal underlying skin [44]. DMF and its utility as a treatment for psoriasis were conceptualised in the late 1950s based on the inaccurate assumption that skin disease was initiated by a TCA cycle biochemical defect and that exogenous administration of FA, an intermediate TCA cycle product, could re-establish its balance [45]. Whilst free FA is poorly absorbed by the GI tract [46], Altmeyer et al. [47] demonstrated that its ester derivatives, MMF and DMF, were beneficial to treat psoriasis in 100 patients in a randomised double-blind study. Following this, an FA mixture comprised largely of DMF (60%) and three ethyl hydrogen fumarates (calcium, magnesium and zinc salts of monoethyl fumarate (MEF)) were approved for the treatment of moderate and severe forms of psoriasis under the brand name Fumaderm^®^ [48]. DMF was ultimately discovered to be the bioactive component in the preparation [49] since MEF salts alone exerted no significant clinical efficacy [50]. More recently, a DMF-only formulation, Skilarence^®^, was approved by the European Medicines Agency as it provided comparable efficacy and safety profiles to that of Fumaderm^®^ in a Phase III double-blind, placebo-controlled trial with 671 patients (NCT01726933) [40]. While the aetiology of psoriasis is still not fully elucidated, it is classified as an immune-mediated disorder owing to a combination of genetic, immunological and environmental factors [51]. As the complex interaction between these three immunomodulatory factors has become better characterised [52,53,54], the primary mode of action (MOA) of DMF was established as immunomodulation [55,56]. In light of this, as well as DMF’s robust efficacy [39,40] and acceptable safety [39,57] profile, the prospect of utilising FAEs for other diseases that share an immune-mediated origin with psoriasis, such as MS, was proposed.

### 2.2. DMF for Multiple Sclerosis (MS)

Owing to their success in treating psoriasis, Schilling et al. [58] investigated FAEs in the experimental autoimmune encephalomyelitis mouse model, which reproduces several of the typical features of human MS. This study revealed therapeutic effects of FAEs prompting further investigation and clinical studies with DMF, specifically in MS patients. MS is a chronic inflammatory demyelinating disorder of the central nervous system characterised by dysregulation of innate and adaptive immune responses [59]. Relapsing-Remitting MS (RRMS) is the most common MS subtype, characterised as a clear increase in new or existing neurological symptoms followed by periods of incomplete or complete recovery, termed remissions. The first exploratory clinical trial of FAE in RRMS took place in 2006 where Fumaderm^®^ was given to 10 patients [60]. Although this study was a small, open-label pilot study, FAEs induced promising improvements in both radiological and clinical parameters and paved the way for continuing clinical trials with DMF. BG-12, an orally administered, enteric-coated micro tablet slow-release preparation of DMF [38] (marketed as Tecfidera^®^), was formulated to reduce GI-related side effects including diarrhoea and nausea and trialled in one Phase II [2] and two subsequent Phase III trials [1,61]. In 2008, the Phase IIb clinical trial (NCT00168701) established efficacy and safety of several different dosages of the delayed release formulation [2]. BG-12 reduced the number of new lesions as well as decreased annualised relapse rates compared to the placebo [2]. These favourable results prompted further long-term Phase III studies in a larger group of patients with RRMS. In 2012, the Determination of the Efficacy and Safety of Oral Fumarate in RRMS (DEFINE; NCT00420212) [1] and Comparator and an Oral Fumarate in RRMS (CONFIRM; NCT00451451) [61] studies were published. Over their 2-year duration, these multicentre, placebo controlled, double blind clinical trials proved that daily treatment of BG-12 decreased the rate of disease progression, number of lesions (observed via magnetic resonance imaging) and significantly reduced the proportion of relapsing patients [1,61]. Although the safety and tolerability of DMF were deemed acceptable in these trials, the incidence of side effects such as flushing, as well as GI events including diarrhoea, upper abdominal pain, vomiting and nausea, were commonly reported by patients in the first month of treatment. Thereafter, the number of side effects reported by patients reduced significantly [62]. While flushing is associated with increased prostaglandin production through key MOA agonism of HCAR2, GI symptoms are the primary reason for treatment discontinuation [63,64] and attributable to the action of major metabolite methanol on small intestine mucosa [63]. Progress toward improved formulations/molecules to minimise GI-related side effects, whilst maintaining the efficacy of DMF have recently been made.

Diroximel fumarate (DRF) (marketed as Vumerity^®^), is a novel orally administered FAE, approved in late 2019 for the treatment of relapsing forms of MS. Approval was based on data from an open-label, Phase III study (NCT02634307) that demonstrated long-term safety and efficacy [63] in addition to another study (NCT03093324) that compared Vumerity^®^ against Tecfidera^®^ [13]. The proposed mechanism through which DRF could improve gut tolerability compared to DMF was through substitution of DMF’s methanol leaving group with inert 2-hydroxyethyl succinimide (HES) [63]. Upon oral administration, DMF is cleaved by gut esterases to yield the major metabolites MMF and methanol; whereas DRF undergoes esterase cleavage to yield major metabolites MMF and HES as well as minor metabolites RDC-8439 and methanol [63]. Otherwise, DRF shares a comparable pharmacokinetic profile to DMF [65]. DRF was shown to be well-tolerated with fewer GI reactions in comparison to DMF in the study [13]. At therapeutic doses, DRF and DMF yielded bioequivalent production of the active metabolite MMF, which has been proposed to exert most of the efficacy imparted by FAEs in MS patients [13]. Another DMF bioequivalent, Bafiertam^™^ (MMF) was recently approved (April 2020) for RRMS. Since a lower dose of Bafiertam^™^ is required compared to Tecfidera^®^ to produce equivalent plasma MMF levels, reduced adverse effects have been purported. Although Bafiertam™ was not independently evaluated in clinical trials with MS patients, it has been approved based on DMF’s efficacy in RRMS. It is possible that FAEs might also be useful to treat diseases that, like psoriasis and MS, are associated with oxidative stress and/or inflammation: this has been an area of intense clinical and pre-clinical research over the past decade.

## 3. Clinical Trials (Novel Indications)

FAEs including DMF/MMF and DRF proved to be valuable therapeutics against psoriasis and MS. In particular, DMF was shown to activate the intrinsic cellular response to oxidative stress [66] and counteract cytotoxic insult [67] to promote cell survival. For these reasons, DMF and its esters are purported to afford therapeutic benefits in other diseases complicated by oxidative stress and inflammation, such as amyotrophic lateral sclerosis (ALS). ALS is a neurodegenerative, inflammatory disorder that results in progressive paralysis and death from respiratory failure within 2–3 years [68]. Although the aetiology of ALS lies in the progressive degeneration of motor neurons, innate immune responsivity has been implicated in the initiation and progression of the disease [69,70]. Reduced levels of regulatory T cells in conjunction with increased macrophage activation [70] and pro-inflammatory effector T cells and cytokines [71] are indicators of rapid disease progression in ALS. Tecfidera^®^ (DMF) increased the ratio of anti-inflammatory to pro-inflammatory T-cell subsets [72,73] and reduced pro-inflammatory T cells in RRMS [72], resulting in enhanced anti-inflammatory activity and neuroprotection. Based on these encouraging results, Vucic et al. (2020) are currently undertaking a Phase II randomised, double blind clinical trial (ACTRN12618000534280) to assess the efficacy and safety of Tecfidera^®^ in patients with ALS. The trial commenced in 2018 with anticipated results in late 2020 or early 2021 [74].

A Phase II clinical trial for Cutaneous T Cell Lymphoma (CTCL) is also currently underway with a projected end date of late 2021 (NCT02546440). CTCLs are a heterogeneous group of non-Hodgkin’s lymphomas characterised by cutaneous infiltration of malignant monoclonal T lymphocytes [75]. Previous pre-clinical trials proposed that DMF inhibits tumour growth and distant metastasis, and restores sensitivity of CTCL cells towards apoptosis by down-regulating elevated nuclear factor κ-light-chain-enhancer of activated B cells (NF-κB) [76]. NF-κB is a transcription factor protein complex involved in inflammation, immune responsiveness and apoptosis [77]. Since apoptosis re-sensitisation was only detected in tumour cells (and not healthy lymphocytes) [76], DMF could be an attractive disease modifying co-treatment candidate in CTCL due to its inherent lack of toxicity and favourable safety profile.

Obstructive sleep apnoea (OSA) is a common disorder that involves collapse of the upper airway during sleep, which results in low blood oxygen levels and sleep disruption when left untreated. OSA increases the risk of cardiovascular-related complications including high blood pressure, heart disease and stroke [78]. OSA has been consistently linked to inflammation, with elevated pro-inflammatory cytokines and adhesion molecules in OSA patients [79,80,81], where systemic hyperinflammation caused by other complications increases the severity of OSA [82]. Intermittent hypoxia associated with OSA is acknowledged as a driver of inflammation [83,84], and at least partially mediated by the activation of NF-κB [85]. FAEs inhibit NF-κB through activation of both Nrf2 and HCAR2, and consequently, the secretion of pro-inflammatory molecules [17]. Studies have revealed a formal association between the use of immunomodulatory/immunosuppressive agents and reduced OSA severity and/or frequency [82,86]. As such, a Phase II randomised, placebo-controlled clinical trial was initiated (NCT02438137) to determine whether DMF was effective for the treatment of OSA. In this yet peer-reviewed study, DMF attenuated disease severity suggesting that OSA may be responsive to agents that regulate immune signalling pathways. Suppression of systemic inflammation via NF-κB reduction was hypothesised as the likely mediator of DMF’s efficacy in OSA.

Whilst DMF demonstrated efficacy in numerous clinical trials, it is important to highlight that some studies failed to show therapeutic efficacy or were abandoned or terminated/withdrawn. A Phase II study that commenced in 2008 trialled DMF (BG-12) in patients with active rheumatoid arthritis. Although DMF activated the Nrf2 pathway eliciting a downward trend for some inflammatory markers and cytokines, it did not significantly modify the pre-defined end-point outcome measures (unpublished clinical trial data; NCT00810836). Another study that investigated the therapeutic potential of DMF in systemic sclerosis-associated pulmonary arterial hypertension (NCT02981082) was terminated due to low recruitment numbers. Similarly, due to lack of funding, a trial investigating the efficacy, safety and tolerability of DMF in patients with chronic lymphocytic leukemia/small lymphocytic lymphoma, was terminated in 2019. Other completed studies include the use of DMF in glioblastoma multiforme (NCT02337426) and cutaneous lupus erythematosus (NCT01352988) completed in 2017 and 2014, respectively, with no results published.

## 4. Pre-Clinical Trials

Recently, Jadeja et al. [87] published a detailed overview of the pre-clinical literature evaluating FAEs for the prevention and treatment of diseases in which oxidative stress and/or inflammation are prominent (other than psoriasis and MS). As such, the pre-clinical studies of FAE will only be briefly discussed here.

### 4.1. GI/Digestive Tract Indications

Inflammatory bowel disease (IBD) is a collection of disorders that are characterised by dysfunction of mucosal immune response and abnormal cytokine production, which contributes to chronic inflammation in the digestive tract [7]. Although there is a range of treatment options available for diseases associated with IBD, given the complex aetiology, the demand for novel and/or improved therapeutic strategies remains high. Since there is a well-established association between IBD development/progression and inflammation, several groups have sought to investigate the efficacy of FAE in the treatment of GI-associated disorders. In most instances, experimental rodent models of colitis have been used to demonstrate the efficacy of DMF for mitigating colon injury, pro-inflammatory cytokine and adhesion molecule production and NF-κB signalling [7]. Furthermore, DMF exerts efficacy in IBD through induction of the Nrf2-mediated antioxidant response involving increased superoxide dismutase (SOD) expression to reduce lipid peroxidation [7,36]. MMF has also been shown to suppress or completely prevent gastric ulceration in rats [88] highlighting FAEs as potent gastro-protective agents. Together, studies like these surprisingly indicate that DMF/MMF and other FAEs may be of clinical benefit for GI indications, despite themselves eliciting GI side effects particularly in the initial stages of treatment [2,57,63,89]. The beneficial effects of FAEs appear to also extend beyond the GI tract. Both MMF [90] and DMF [91] exhibited hepatoprotective effects in various models of hepatotoxicity by reducing inflammation and oxidative stress. Larger studies to further develop/refine the efficacy of FAE formulations in GI and hepatic conditions are warranted to establish their clinical potential.

### 4.2. Neurological Indications

The brain is especially sensitive to perturbations caused by oxidative and/or inflammatory stress. In fact, these factors are central to the pathogenesis of several neurodegenerative diseases including Alzheimer’s disease (AD) [33,92], Parkinson’s disease (PD) [93], Huntington’s disease (HD) and intracerebral haemorrhage (stroke) [94]. Concomitantly, these diseases are also underscored by pathological mitochondria and perturbations in energy homeostasis (reviewed in [95,96]). Therefore, therapies that enhance anti-oxidative potential, counter stressors and promote mitochondrial pool maintenance and function may be of clinical value. FAE can induce Nrf2 signalling and cellular defence machinery against oxidative stress [67], which should therefore provide some neuroprotection in disorders such as AD, PD and HD. In mouse models of these diseases, DMF reduced damage and degeneration to preserve neuronal populations [10,33,97,98] by upregulating Nrf2-dependent antioxidant genes [10,97] and suppressing NF-κB-mediated inflammation [10,69,70,97]. These molecular adaptations attenuated clinical symptoms including motor impairment [69], long-term memory deficits [70] and increased overall survival rates [69]. One downstream response to Nrf2 activation is mitochondrial biogenesis through key regulators, peroxisome proliferator-activated receptor gamma coactivator 1-alpha (PGC1α) and mitochondrial transcription factor A (TFAM) [99]. Pharmacological up-regulation of mitochondrial biogenesis and turnover has shown therapeutic value in mouse models of PD and HD [95] and mitochondrial function stimulants have shown similar pre-clinical benefits in AD [96]. Since DMF can stimulate mitochondrial substrate flux, anaplerosis and energy production during its endpoint metabolism (i.e., fumarate) in the mitochondrial TCA cycle, as well as activate Nrf2, it could exert additional therapeutic activity in this regard. DMF was also beneficial in ischemia-related pathogenesis such as that elicited through stroke, where neuroprotection and neuronal survival can be enhanced to avert ischemic injury. In mouse [100,101] and rat [102] models of stroke, DMF reduced ischemia-associated inflammation [100,101,102], stabilised the blood brain barrier [100,101], prevented cerebral oedema [71,101,102], enhanced neurological recovery [71,101,102] and importantly, ameliorated neurological deficits and improved hematoma resolution even when administered as late as 24 h after the haemorrhage event [102]. These pre-clinical studies highlight the clinical potential for FAEs to be repurposed for neurodegenerative and ischemia-related disorders of the brain.

### 4.3. Cancer Related Indications

Accumulating evidence suggest that DMF exerts anti-tumorigenic properties in several types of cancers. DMF was first implicated in the inhibition of cancer cell growth in the early 2000s when its effect on melanoma growth and metastasis was evaluated in animal models and human melanoma cell lines [103]. This work was supported by the discovery that DMF reduces cell invasion and metastasis of melanoma cells by inhibiting pro-metastatic proteases such as the matrix metalloproteinases (MMPs) [104,105], as well as by inducing apoptosis and cell cycle inhibition [105,106]. More recently, Takeda et al. [105] proposed that DMF prolongs survival in melanoma mouse models and exerts its anti-metastatic efficacy through suppression of nuclear translocation of NF-κB. NF-κB is constitutively active in several types of cancer [107,108,109]. Especially in breast cancer, NF-κB signalling enhances tumour cell survival, migration, invasion, angiogenesis and resistance to anti-cancer therapy [110,111]. NF-κB is thus a prognostic indicator of aggressive breast cancers [111]. Consistent with its anti-NF-κB activity, DMF inhibited mammosphere formation (a functional measure of cancer stem cell properties), cell proliferation and xenograft tumour growth [110], providing proof-of-concept evidence for the clinical potential of DMF in breast cancer therapy. Other cancer cell lines, including colorectal cancer [37], cervical cancer [112], lung adenocarcinoma [113] and pancreatic carcinoma [113] are also sensitive to DMF-induced cytotoxicity. These studies suggest that FAE could be broadly utilised as an anti-cancer therapy.

### 4.4. Cardiovascular Indications

The role of oxidative stress in the aetiology of vasculopathy and hypertension is well-documented [114]. Excessive ROS production diminishes nitric oxide bioavailability, leading to increased vasoconstriction and damage to the vasculature by inducing inflammation and fibrosis [115]. Although the specific MOA remains to be determined, the cardioprotective effect of fumarate has been associated with its metabolism to succinate through both oxidative and reductive pathways. This was confirmed by Laplante et al. [116] where metabolic fluxes in fumarate-perfused rat hearts were studied. Since then, some evidence has emerged to suggest that DMF may be useful for cardiovascular related indications. Grzegorzewska et al. [34] demonstrated in mouse models of pulmonary arterial hypertension and lung fibrosis that DMF reduced inflammation, oxidative damage and fibrosis. DMF also attenuated abnormal remodelling after acute vascular injury in rat carotid arteries [35]. Whilst these studies suggest that FAE might be beneficial for patients with vascular diseases, more research is warranted to establish DMF efficacy for these indications.

## 5. Novel Applications

**Figure 1:**
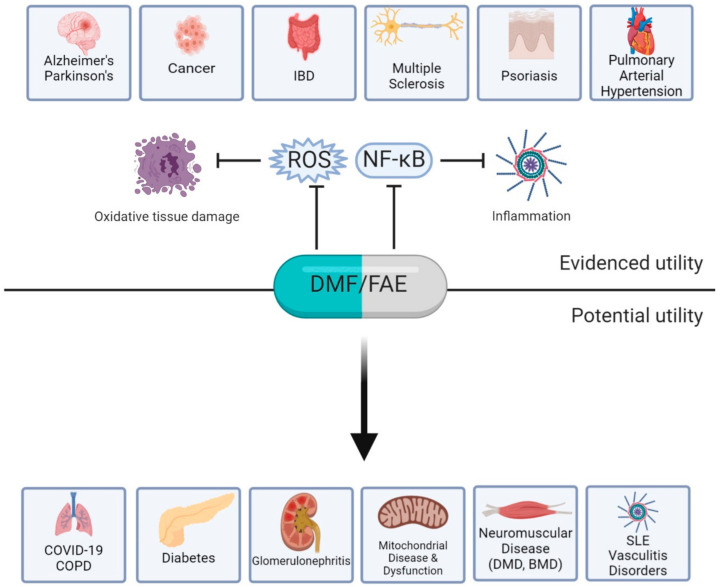
Approved and evidenced utility of dimethyl fumarate (DMF) and fumaric acid esters (FAE) versus potential utility in other diseases that are underscored by oxidative stress and hyperinflammation. DMF and FAE can exert their effects by enhancing cytoprotective and anti-inflammatory responses. This is proposed to lead to the reduction of reactive oxygen species (ROS), which in turn prevents oxidative tissue damage. DMF also inhibits nuclear factor κ-light-chain-enhancer of activated B cells (NF-κB), which subsequently reduces pro-inflammatory cytokine production and immune cell deviation and ultimately leads to reduced inflammation. BMD: Becker Muscular Dystrophy; COPD: chronic obstructive pulmonary disease; DMD: Duchenne Muscular Dystrophy; IBD: inflammatory bowel disease; SLE: systemic lupus erythematosus. Bar heads = inhibitory effect; Arrow heads = potential beneficial effect. Created with BioRender.com.

The combined Nrf2-inducing and immune-modulatory properties of FAE and their robust bioavailability have enabled drugs such as DMF and MMF to be versatile across pathologies in a range of body systems (summarised in Figure 1). Thus, the potential clinical impact of FAE therapy is high and particularly broad. The evidence provided in this review, as well as by others [45,49,87], compellingly demonstrates that, since oxidative stress and inflammation are inextricably linked, FAEs have the potential to positively influence diseases characterised by both of these factors. This could include chronic and progressive neuromuscular diseases such as Duchenne and Becker Muscular Dystrophy; mitochondrial diseases such as Friedreichs’s Ataxia as well as other diseases featuring mitochondrial dysfunction; respiratory-related diseases including novel coronavirus (COVID-19) since it has recently been proposed that severely affected patients manifest a cytokine storm syndrome [117] and treatment of hyperinflammation may reduce mortality rate; renal diseases including glomerulonephritis and; metabolic disorders such as diabetes. Less potent Nrf2 activators (sulforaphane [26,118], curcumin [25,119,120] and resveratrol [27,28,121]) have been developed, tested and demonstrated for use in some of these indications, however do not appear to exert the same influences on innate and adaptive immunity as FAEs. Since FAEs such as DMF are approved, affordable, widely available and clinically well characterised, they are appealing candidates for expanded clinical development. As such, exploratory trials of FAE therapies in a variety of other diseases is warranted (Figure 1).

## 6. Conclusions

FAEs are currently approved for several indications including psoriasis and RRMS, due to their anti-inflammatory, anti-oxidative and immunomodulatory properties and are investigated for many other indications. FAEs are drugs with broad clinical utility and favourable safety profiles, which could be quickly expanded to treat other diseases with unmet clinical needs through drug repurposing strategies.

## Figures and Tables

**Table 1:** Summary of fumaric acid esters either approved for human use, previously tested or currently examined in human/animal trials.

Status	Compound	Disease	Phase of Trial
Approved for Human Use	Dimethyl Fumarate	Multiple Sclerosis	Approved
		Psoriasis	Approved
	Diroximel Fumarate	Multiple Sclerosis	Approved
	Monomethyl Fumarate	Multiple Sclerosis	Approved
Human Trials	Dimethyl Fumarate	Amyotrophic Lateral Sclerosis	Phase II
		Cutaneous T Cell Lymphoma	Phase II
		Glioblastoma Multiforme	Phase II
		Obstructive Sleep Apnoea	Phase II
		Rheumatoid Arthritis	Phase II
Pre-clinical Animal Trials	Dimethyl Fumarate	Breast Cancer	Pre-clinical
		Colitis	
		Melanoma	
		Pancreatitis	
		Parkinson’s Disease	
		Sickle Cell Disease	
	Monomethyl Fumarate	Gastric Ulcer	
		Sickle Cell Retinopathy	
